# Review of the green lacewing genus *Chrysacanthia* Lacroix with a new species from Nigeria (Neuroptera, Chrysopidae)

**DOI:** 10.3897/zookeys.517.9705

**Published:** 2015-08-12

**Authors:** Shaun L. Winterton, Stephen J. Brooks

**Affiliations:** 1 California State Collection of Arthropods, California Department of Food & Agriculture, Sacramento, California, USA; 2 Department of Entomology, The Natural History Museum, London, Great Britain

**Keywords:** Afrotropical, Neuroptera, Belonopterygini

## Abstract

The genus *Chrysacanthia* Lacroix (Chrysopidae: Belonopterygini) is reviewed and a new species is described from Nigeria. With the addition of the new species described herein, the genus contains four Old World species known from Madagascar, Nigeria, India, Thailand and China.

## Introduction

Chrysopidae (green lacewings) represent the second largest family of Neuroptera, with approximately 80 genera comprising over 1200 species in found throughout all major biogeographical regions, particularly the tropics (Brooks and Barnard 1990). Chrysopids are divided into three extant subfamilies, Apochrysinae Handlirsch, Nothochrysinae Navás and Chrysopinae Schneider. Apochrysinae contains six pantropical genera of large, delicate lacewings frequently found in densely forested habitats (Kimmins 1952; Winterton and Brooks 2002). The subfamily Nothochrysinae comprises nine extant genera world-wide (plus numerous fossil taxa) with many species that exhibit plesiomorphic characteristics (Adams 1967; Brooks and Barnard 1990; Adams and Penny 1992). The majority of the generic and species-level diversity in green lacewings is found in the subfamily Chrysopinae, which includes approximately 97% of all living species. This subfamily is additionally subdivided into four tribes: Belonopterygini Navás, Chrysopini Schneider, Leucochrysini Adams and Ankylopterygini Navás (Brooks and Barnard 1990; Winterton and de Freitas 2006). Belonopterygini (formerly Italochrysini Hölzel) represents one of the smallest of these tribes, with 14 genera distributed in all major biogeographic regions. Most individuals in this tribe are relatively large and robust chrysopids, frequently having dark yellow to brownish-tan colouration and dark markings on the body.

**Figure 1.**
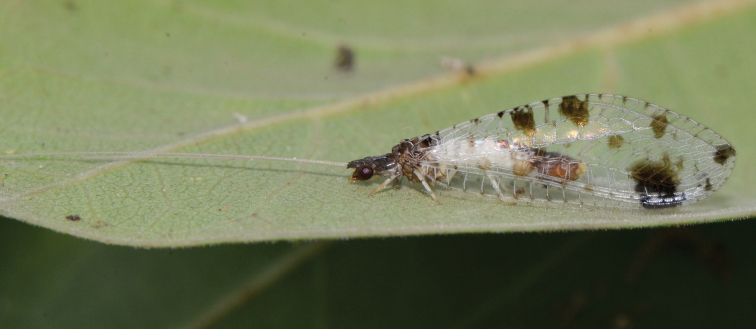
*Chrysacanthia esbeniana* Lacroix. Habitus, India (Photo: Poorani Janakiraman).

The distinctive Old World genus *Chrysacanthia* Lacroix is reviewed and *Chrysacanthia iwo* sp. n. described from Nigeria. All previously described species placed in *Chrysacanthia* were originally the bases for monotypic genera. The type species *Chrysacanthia esbeniana* Lacroix, (Fig. 1) was described from India (Lacroix 1923). Fraser (1951) subsequently described a second species in the genus *Nesochrysa* Fraser from Madagascar (Fig. 3B) and Yang and Yang (1991) (Fig. 3C) described a third species from China in the genus *Xanthochrysa* Yang & Yang. Brooks and Barnard (1990) and Brooks (1997) consolidated these genera into one genus containing three distinct, but clearly closely related, species with a highly disparate distribution. The genus *Chrysacanthia* is diagnosed relative to this expanded species composition and a key to species of *Chrysacanthia* presented.

## Materials and methods

Terminology follows Tjeder (1966) and Brooks and Barnard (1990). Genitalia were macerated in 10% KOH to remove soft tissue, then rinsed in distilled water and dilute glacial acetic acid, dissected in 80% ethanol and subsequently stained with a solution of Chlorazol Black in 40% ethanol. The dissected genitalia were placed in glycerine in a genitalia vial mounted on the pin beneath the specimen.

## Taxonomy

### Tribe Belonopterygini Navás

#### Chrysacanthia

Taxon classificationAnimaliaNeuropteraChrysopidae

Lacroix

Chrysacanthia Lacroix, 1923: 120. Type species: *Chrysacanthia esbeniana* Lacroix, 1923: 121, by monotypy.
Nesochrysa Fraser, 1951: 29. Type species: *Nesochrysa varicella* Fraser, 1951: 29, by monotypy.
Xanthochrysa Yang & Yang, 1991: 207. Type species: *Xanthochrysa hainana* Yang & Yang, 1991: 207, by monotypy.

##### Diagnosis.

Small to medium sized lacewings: forewing length: 14–17 mm; hindwing length: 12–14 mm. Wings with dark markings, particularly on forewing; four rings of setae on flagellomeres; palpi rounded apically; pronotum relatively broad; Sc and R widely separated; Sc terminating well before wing apex; cell im short, broad and ovate (not quadrangular); m2 relatively short; gradates in two series; inner gradate series meeting Psm; male veins not crassate basally; c1 1.5–2.0 times length of c2; abdomen whitish-coloured, sternite 7 dark, tergites 4–8 polished black-brown; male 9^th^ tergite+ectoproct yellowish-brown, lacking elongate processes; parameres elongate, extending beyond apex of abdomen; gonarcus broad with elongate gonocornua; gonosaccus with a few dispersed gonosetae; female sternite 7 with posteromedial swelling; praegenitale distinct on sternite 7.

##### Included species.

*Chrysacanthia esbeniana* Lacroix, *Chrysacanthia hainana* (Yang); *Chrysacanthia varicella* (Fraser); *Chrysacanthia iwo* sp. n.

##### Distribution.

Afrotropical: Nigeria, Madagascar; Oriental: China, India, Thailand.

##### Comments.

**Figure 2.**
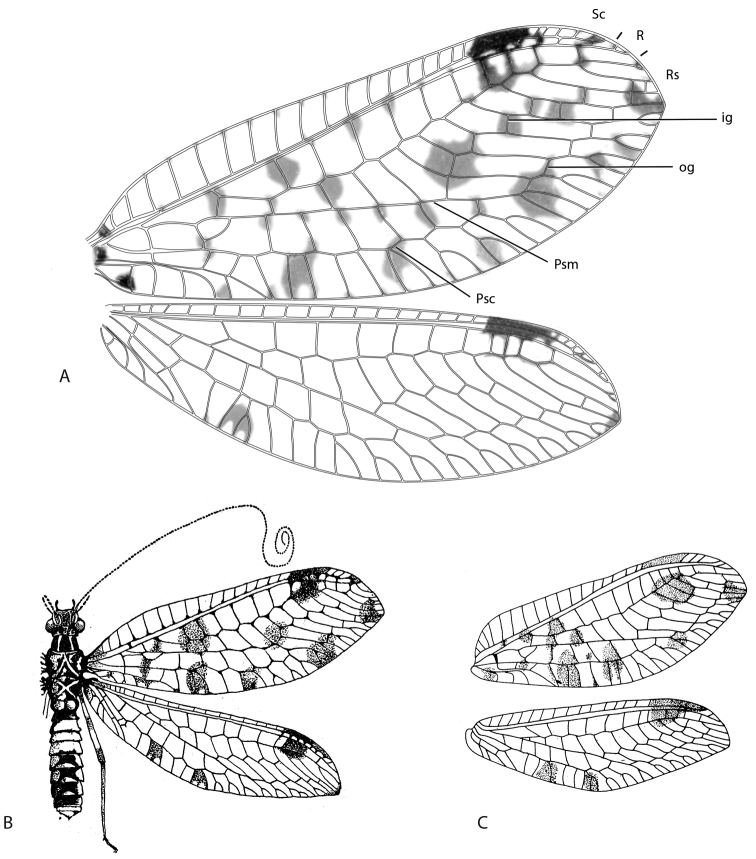
*Chrysacanthia* spp. **A** *Chrysacanthia iwo* sp. n., forewing and hind wing (Forewing length: 15.0 mm) **B** *Chrysacanthia varicella* (Fraser), body and wings (after Fraser 1951: figure 8) **C** *Chrysacanthia hainana* (Yang & Yang) forewing and hind wing (after Yang and Yang 1991: figure 2). Abbreviations: *ig*, inner gradate series; *psc*, pseudocubital vein; *psm*, pseudomedial vein; *og*, outer gradate series (drawings not to scale; vestiture omitted).

**Figure 3.**
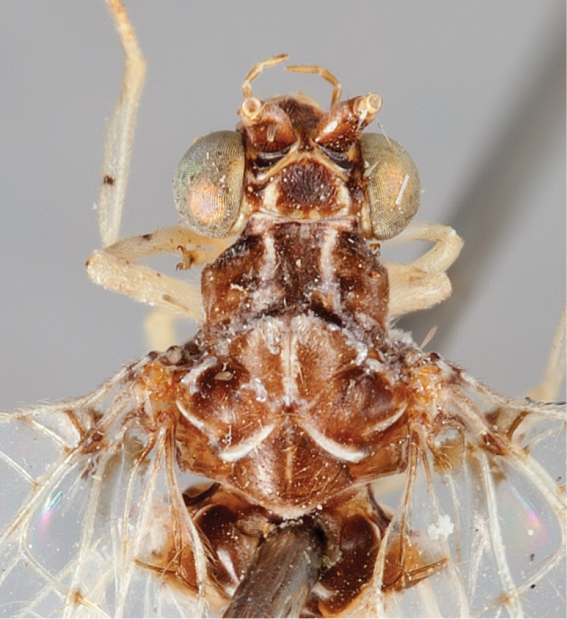
*Chrysacanthia iwo* sp. n. male head and thorax [abdomen dislodged during dissection].

*Chrysacanthia* is a distinctive genus is easily recognized by the dark head and thorax, with cream-coloured abdomen with black tergites posteriorly, and dark markings on the wings (Figs 1–3). Fewer than 10 specimens of this genus have been collected and the four species described are disparately distributed throughout the Oriental and Afrotropical regions. In the Afrotropical region *Oyochrysa* Brooks is superficially similar with extensive wing and body markings, but it is larger with distinctly different male genitalia.

### Relationships among Belonopterygini genera and position of *Chrysacanthia*

Few comprehensive estimates of green lacewing higher-level phylogeny have been published, either based on morphology (*e.g.*, Brooks and Barnard 1990; Winterton and Brooks 2002) or on DNA sequence data (*e.g.*, Winterton and de Freitas 2006; Haruyama et al. 2008). Consequently detailed knowledge of subfamilial, tribal or generic relationships within Chrysopidae remains poorly understood. This is particularly true of our understanding of Belonopterygini phylogeny. Brooks and Barnard (1990) suggested that the tribe represented the sister to all other Chrysopinae, as members display numerous plesiomorphic characteristics. They also identified a number of genitalic features shared by both Belonopterygini and Leucochrysini which support a sister group relationship between the two tribes, a hypothesis also supported (along with Ankylopterygini) by DNA sequence data (Winterton and de Freitas 2006). Belonopterygini are differentiated from other Chrysopinae by (1) the relative distal placement of the basal subcostal crossvein, (2) broad pronotum, (3) thick apical palpal segment, (4) relatively broad flagellomeres, (5) wing cell c1 longer than c2, (6) male terminalia typically with parameres, and (7) female terminalia with praegenitale usually present (Brooks 1984; Brooks and Barnard 1990). In Chrysopidae, when the parameres articulate with the gonarcus they are referred to as entoprocesses (Adams 1962; Tjeder 1966). The homology of these structures is not confirmed in all taxa though, as in at least *Abachrysa* Banks, both are present (Brooks and Barnard 1990). In Belonopterygini the parameres are a distinctive component of the male genitalic armature; they do not articulate with the gonarcus and are partially fused medially. The gonarcus is often arched and in some genera non-articulating lateral processes termed gonocornua are present (*e.g*., *Nesochrysa* Navás) (Brooks and Barnard 1990), presumably analogous to entoprocesses. Generic concepts within Belonopterygini are largely defined (among other characters) based on the shape of the wing cell *im* and genitalic complement (*e.g*., presence/absence of parameres, entoprocesses and praegenitale). Although lost in some genera, praegenitale are only found in Belonopterygini and are considered an apomorphy of the group (Brooks and Barnard 1990).

As previously stated, generic relationships are largely unknown in Belonopterygini, yet certain patterns are evident which suggest likely groupings of genera. Genus groups within the tribe can be identified based on the complement of male genitalic structures (Brooks and Barnard 1990). Along with the presence and absence of parameres, the gonarcus may have articulating entoprocesses or non-articulating gonocornua present. In the New World there are three genera, *Nacarina* Navás, *Abachrysa* and the enigmatic type genus *Belonopteryx* Gerstaecker. These genera appear to be closely related and likely form a clade sister to the remaining Belonopterygini (Brooks and Barnard 1990). Among other shared characteristics, *Belonopteryx* and some species of *Nacarina* lack parameres and a praegenitale, structures typically found in the male and female terminalia (respectively) of most other genera in this tribe. Brooks and Barnard (1990) described the genus *Evanochrysa* Brooks & Barnard from the Oriental region and suggested that it was closely related to *Nacarina* based characteristics such as this lack of male parameres as well as the presence of gonosetae.

The greatest generic diversity in Belonopterygini is in the Old World, principally the Afrotropical region, where genera such as *Oyochrysa*, *Dysochrysa* Tjeder, *Turnerochrysa* Kimmins, *Chrysaloysia* Navás and *Nesochrysa* are endemic. Besides *Evanochrysa*, the Oriental and Eastern Palaearctic regions contain endemic genera such as *Stigmachrysa* Navás and *Nodochrysa* Banks. There is only a single endemic genus (*Calochrysa* Banks) in the Australasian region. Two genera that are widely distributed throughout the Old World are *Italochrysa* Principi and *Chrysacanthia*. With approximately 100 species, many more than all other genera combined, *Italochrysa* is the dominant genus of Belonopterygini and occurs throughout the Afrotropical, Palaearctic, Oriental and Australasian regions (Tjeder 1966; Brooks and Barnard 1990; New 1980). Conversely, *Chrysacanthia* contains only four species, but is similarly widely distributed throughout the Afrotropical and Oriental regions.

Among Old World genera, gonocornua are present in genera such as *Nesochrysa*, *Dysochrysa*, *Chrysaloysia*, *Stigmachrysa* and *Chrysacanthia*. Apical lobes on ectoprocts and/or sternite 8+9 in the male suggest a further close relationship among *Nesochrysa*, *Nodochrysa* and *Stigmachrysa*; these lobes are lacking in the other genera in this group with gonocornua. Moreover, the elongate shape of the gonocornua indicates a close relationship among *Chrysaloysia*, *Dysochrysa* and *Chrysacanthia* (Brooks and Barnard 1990). The putative sister genus to *Chrysacanthia* is likely to be *Dysochrysa* based on the shape of the male genitalia and the sub-triangular forewing cell im. *Chrysacanthia* is readily distinguished from *Dysochrysa* based on the extensive wing patterning, which is absent in *Dysochrysa* (Tjeder 1966).

*Turnerochrysa* is a monotypic genus with greatly reduced wing venation associated with its unusually small size for members of this tribe. Relationships of this genus to other Belonopterygini are unclear, but the lack of gonocornua suggests a possible relationship with *Italochrysa* and *Oyochrysa* (Tjeder 1966; Brooks 1984; Brooks and Barnard 1990). *Oyochrysa* and *Italochrysa* are in turn closely related based on the elongate extension of sternite 7 in the female. *Calochrysa* is clearly closely related to *Italochrysa* and is distinguished largely by the presence of a forked vein Cu_2_ in the forewing (New 1980; Brooks and Barnard 1990).

Tauber et al. (2006) and Tauber (2006, 2007) recently proposed the transfer of *Vieira* Navás from Leucochrysini to Belonopterygini based on a series of adult and larval characteristics. *Vieira* is typical of Leucochrysini and does not fit comfortably in Belonopterygini as it is presently defined. Indeed, most of the characters identified supporting this transfer are highly variable even within the tribe, and their value for placement in a phylogenetic context has not been fully tested. Those Belonopterygini characters found in *Vieira* could as easily represent shared plesiomorphies and therefore do not discount a basal position in Leucochrysini. *Vieira* is retained in Leucochrysini for the present until a more comprehensive quantitative phylogenetic analysis can be undertaken on the group.

The immature stages of chrysopids in the tribe Belonopterygini are poorly known, and larvae are documented for only three genera (*Calochrysa*, *Nacarina* and *Italochrysa*) (Weber 1942; Principi 1943, 1946; New 1983, 1986; Tauber and Winterton 2014). Most chrysopid larvae are arboreal generalist predators and many larvae carry a debris packet lodged in elongate setae on their dorsum for camouflage and physical deterrence (Perez de-la Fuente et al. 2012). The larvae are confirmed specialized predators in ant nests in both *Nacarina* and *Italochrysa* (Weber 1942, Principi 1943, 1946; Tauber and Winterton 2014). Belonopterygini larvae have a large number of short hooked setae on the dorsum, presumably enabling carriage of a dense debris packet for physical defence against attack by ants in the nest (Principi 1946; Tauber et al. 2014; Tauber and Winterton 2014). The record by Weber (1942) of *Nacarina* is anomalous in that the larvae did not have a debris packet, suggesting that there is also chemical camouflage to aid in defence against ant attacks. This life history and associated dense debris packet appears specific to Belonopterygini and is considered a synapomorphy for the tribe. Interestingly, the first instar of *Vieira elegans* (Guérin-Méneville), which was described by Tauber et al. (2006), retains much of the chaetotaxy characteristic of non-Belonopterygini tribes.

### Key to species of *Chrysacanthia*

**Table:** 

1	Head and thorax extensively dark brown with pale linear markings (Fig. 3); (Afrotropical)	**2**
–	Head and thorax uniform dark brown, or yellowish brown with distinct brown markings (Oriental)	**3**
2	Wing markings very dark; hind wing with two spots along posterior margin; legs with multiple dark bands on femora (Madagascar)	***Chrysacanthia varicella* (Fraser)** (Fig. 2B)
–	Wing markings relatively pale; hind wing with single spot along posterior margin; legs with femora unmarked (Nigeria)	***Chrysacanthia iwo* sp. n.** (Fig. 2A).
3	Head and thorax mostly dark brown, with some blackish markings; forewing with mark present at base of inner gradate series (India, Thailand)	***Chrysacanthia esbeniana* Lacroix** (Fig. 1).
–	Head yellowish brown with darker markings on vertex and across face; prothorax yellowish medially, dark brown laterally; forewing with mark absent at base of inner gradate series (China)	***Chrysacanthia hainana* (Yang & Yang)** (Fig. 2C)

#### Chrysacanthia iwo sp. n.

Taxon classificationAnimaliaNeuropteraChrysopidae

http://zoobank.org/06FA20A2-2D52-4A41-A3F3-27E83FA33E1D

Figures 2 , 3 , 4

##### Type material.

**Holotype** male, NIGERIA: Osun State: Iwo, 2.iii.1973, cashew leaf, pres. By Comm. Inst. Ent. B.M. 1977-1, BMNH(E) 1201743 (Natural History Museum, London). Type condition: poor, damaged: antennae missing, abdomen and genitalia dissected.

##### Diagnosis.

Head and thorax dark with pale linear markings; hind wing with single mark along posterior margin at pseudomedial crossveins 2–3; femora unmarked.

##### Description.

**Figure 4.**
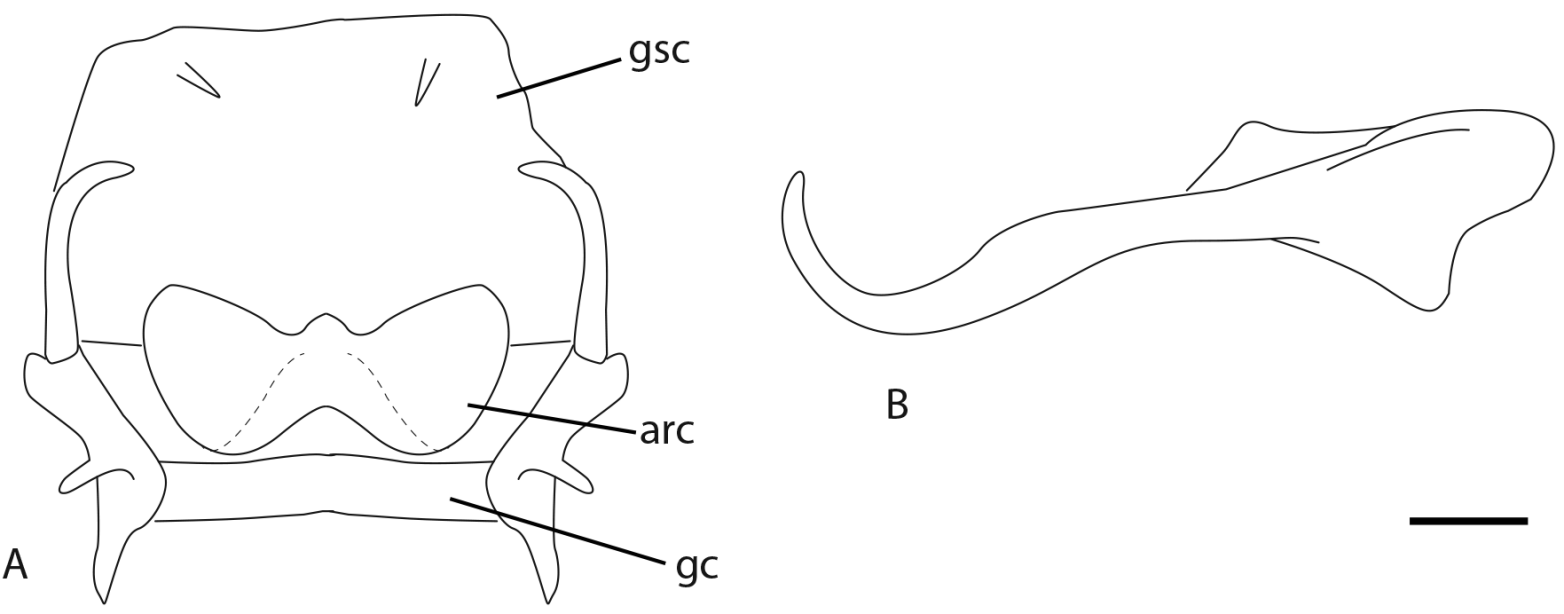
*Chrysacanthia iwo* sp. n., male. **A** gonarcus complex **B** paramere. Abbreviations: *arc*, arcessus; *gc*, gonarcus; *gsc*, gonosaccus. Scale line: 0.2 mm.

Male: Wing length (forewing: 15.0 mm; hindwing: 13.0 mm). Overall colouration very dark brown to black, with cream coloured abdomen with black polished tergites posteriorly and dark markings in wings. Head (Fig. 3). Dark brown with white markings; vertex with pale crescent marking around base of antenna, behind eye and posteriorly along vertex ridge; labrum and gena pale, clypeus with pale suffusion laterally with white band across lower margin; antennal scape dark brown, flagellum colour unknown (missing in specimen); palpi light brown-tan, unmarked. Thorax (Fig. 3). Prothorax dark brown dorsally, cream ventrally, medium length pale setae sparsely distributed; pronotum with two longitudinal mid-dorsal stripes, curving outwards and approximating posterolateral corner, stripes overlain with short dense silver pubescence; mesonotum and metanotum dark brown with pale markings, overlain with pubescence, denser and silvery posteriorly and medially, admixed with sparse pale setae; pleuron dark brown on upper portion, cream on lower portion; legs pale with white setae, tibiae with a narrow dark brown mark at midpoint dorsally; claws pale basally, brownish apically on all legs, claw dilated basally; wings hyaline with extensive makings, especially in forewing; forewing with seven inner gradate crossveins, one set doubled apically, meeting Psm posteriorly; eight outer gradate crossveins, one set doubled apically; two crossveins between Cu_1_ and Cu_2_, 1st posterior marginal crossvein joining wing margin proximal to Cu_2_; hind wing with five inner gradate crossveins, seven outer gradate crossveins; wing hyaline with markings as per Figure 2A, forewing more extensively marked than hindwing; venation mostly white, brown when crossing infuscate areas and at junctions of crossveins with major veins; basal subcostal crossvein dark; pterostigma very dark in both wings; single mark along posterior margin of hind wing and at apex of fore wing. Abdomen. Predominantly white; tergites 4–7 polished black-brown; sternites 7–8+9 brown; sternite 7 with conical posteromedial process; tergite 8 and 9+ectoproct pale. Male terminalia (Fig. 4): Trichobothria *ca.* 35; paramere elongate, upturned apically, not extending beyond apex of abdomen; gonarcus relatively short, broad, with elongate gonocornua; arcessus broad with lateral hook-like process; gonosaccus weakly developed with paired lateral gonosetae.

Female: unknown.

##### Comments.

This Afrotropical species of *Chrysacanthia* is easily differentiated from other species in the genus by the head and thoracic markings (*i.e*., dark brown with pale stripes and arch-like markings), unmarked femora, relatively short paramere, single spot on the posterior margin of the hind wing, and well developed mark at the base of the inner gradate series of the forewing. *Chrysacanthia iwo* sp. n. is known only from the holotype male collected on cashew in Iwo, Nigeria.

Members of this genus are very distinctive based on wing venation and markings on the head and thorax. The Malagasy *Chrysacanthia varicella* was excellently figured by Fraser (1951) (reproduced here; Fig. 2B). This species is very similar to C. iwo sp. n., but can be differentiated by the presence of two wing spots along the posterior margin of the hind wing; in *Chrysacanthia iwo* sp. n. only one spot is present. The Afrotropical species are typified by pale markings on a dark head and thorax, while in the Oriental species are more uniform dark. *Chrysacanthia esbeniana* (India) is distinguished from the other Oriental species, *Chrysacanthia hainana* (China, Thailand), by the presence of a dark spot at the base of the inner gradate series in the forewing (Figs 1, 2C).

##### Etymology.

This new species is named after the type locality, the township of Iwo, SW Nigeria.

## Supplementary Material

XML Treatment for Chrysacanthia

XML Treatment for Chrysacanthia iwo
